# Supplementary material for: Factors Associated with Body Image and Self-Esteem in Mastectomized Breast Cancer Survivors
Source: Int J Environ Res Public Health. 2023 Mar 15;20(6):5154. doi: 10.3390/ijerph20065154 (PMC10048946; doi:10.3390/ijerph20065154)
Supplement: Supplementary file 1 [file ijerph-20-05154-s001.zip › File S1.pdf]

"El saber de mis hijos hará mi grandeza"

PROGRAMA DE ENTRENAMIENTO MUSCULAR PERSONALIZADO ORIENTADO A LA FUERZA EN MUJERES SUPERVIVIENTES AL CÁNCER DE MAMA

PROGRAMA DE ENTRENAMIENTO MUSCULAR EN MUJERES SUPERVIVIENTES AL CÁNCER DE MAMA

ESCALA DE AUTOESTIMA DE ROSEMBERG

Este Test tiene como objetivo evaluar el sentimiento de satisfacción que la persona tiene consigo misma. Por favor, conteste las siguientes frases con la respuesta que considere más adecuada.

A Muy de acuerdo, B de acuerdo, C en desacuerdo, D muy en desacuerdo

	A	B	C	D
Siento que soy una persona de aprecio, al menos, en igual medida que los demás				
Estoy convencido de que tengo cualidades buenas				
Soy capaz de hacer las cosas tan bien como la mayoría de la gente				
Tengo una actitud positiva de mi misma				
En general, estoy satisfecho de mi misma				
Siento que no tengo mucho de que estar orgullosa				
En general, me inclino a pensar que soy una fracasada				
Me gustaría poder sentir más respeto por mi mismo				
Hay veces que realmente pienso que soy una inútil				
A veces creo que no soy buena persona				

ESCALA DE IMAGEN CORPORAL (HOPWOOD)

	Nada	Un Poco	Bastante	Mucho
¿Se ha sentido avergonzada por su apariencia?				
¿Se siente menos atractiva físicamente a causa de su enfermedad o su tratamiento?				
¿Se ha sentido insatisfecha con su apariencia estando vestida?				
¿Se ha sentido menos femenina como consecuencia de su enfermedad o tratamiento?				
¿Le resulta difícil mirarse estando desnuda?				
¿Se ha sentido menos atractiva sexualmente a causa de su enfermedad o tratamiento?				
¿Evita usted a las personas debido a como se siente a causa de su apariencia?				
¿Ha sentido que el tratamiento ha dejado su cuerpo incompleto?				
¿Se ha sentido insatisfecha con su cuerpo?				
¿Se ha sentido insatisfecha con la apariencia de su cicatriz?				
